# K-Ras and p53 mouse model with molecular characteristics of human rhabdomyosarcoma and translational applications

**DOI:** 10.1242/dmm.049004

**Published:** 2022-02-17

**Authors:** Kengo Nakahata, Brian W. Simons, Enrico Pozzo, Ryan Shuck, Lyazat Kurenbekova, Zachary Prudowsky, Kshiti Dholakia, Cristian Coarfa, Tajhal D. Patel, Lawrence A. Donehower, Jason T. Yustein

**Affiliations:** 1 Texas Children's Cancer and Hematology Centers and The Faris D. Virani Ewing Sarcoma Center, Baylor College of Medicine, Houston, TX 77030, USA; 2 Center for Comparative Medicine, Baylor College of Medicine, Houston, TX 77030, USA, USA; 3 Translational Cardiomyology Laboratory, Stem Cell Research Institute, Stem Cell Biology and Embryology Unit, Department of Development and Regeneration, KU Leuven, Leuven 3000, Belgium; 4 Cancer and Cell Biology Program, Baylor College of Medicine, Houston, TX 77030, USA; 5 Department of Molecular and Cellular Biology, Baylor College of Medicine, Houston, TX 77030, USA; 6 Dan L. Duncan Cancer Comprehensive Center, Baylor College of Medicine, Houston, TX 77030, USA

**Keywords:** GEMM, Rhabdomyosarcoma, K-Ras, Syngeneic models

## Abstract

Rhabdomyosarcoma (RMS) is the most common soft tissue sarcoma in children, with overall long-term survival rates of ∼65-70%. Thus, additional molecular insights and representative models are critical for identifying and evaluating new treatment modalities. Using MyoD-Cre-mediated introduction of mutant K-Ras^G12D^ and perturbations in p53, we developed a novel genetically engineered mouse model (GEMM) for RMS. The anatomic sites of primary RMS development recapitulated human disease, including tumors in the head, neck, extremities and abdomen. We confirmed RMS histology and diagnosis through Hematoxylin and Eosin staining, and positive immunohistochemical staining for desmin, myogenin, and phosphotungstic acid–Hematoxylin. Cell lines from GEMM tumors were established with the ability to engraft in immunocompetent mice with comparable histological and staining features as the primary tumors. Tail vein injection of cell lines had high metastatic potential to the lungs. Transcriptomic analyses of p53^R172H^/K-Ras^G12D^ GEMM-derived tumors showed evidence of high molecular homology with human RMS. Finally, pre-clinical use of these murine RMS lines showed similar therapeutic responsiveness to chemotherapy and targeted therapies as human RMS cell lines.

## Abstract (precis)

**Editors' choice:** We developed a new conditional genetically engineered mouse model of rhabdomyosarcoma (RMS) with homologous molecular signature to human RMS that provides valuable pre-clinical models for evaluating novel therapies.

## INTRODUCTION

Rhabdomyosarcoma (RMS) is the most common soft tissue sarcoma in children. Despite improvements in patient survival rates over the past few decades, over one-third of RMS patients still succumb to the disease. Therefore, identification of additional effective therapeutic regimens is crucial towards improving the outcome for these patients. RMS can be categorized histologically into alveolar RMS (ARMS) and embryonal RMS (ERMS) (Yohe et al., 2019). The alveolar subtype has a distinct alveolar architecture, with aggregates of small round undifferentiated cells separated by dense hyalinized fibrous septa. At the center of the tumor, the clusters are arranged loosely and therefore they look like an alveolar. The embryonal subtype resembles microscopically the various stages of muscle development from poorly differentiated round tumor cells to well-differentiated cells, with cross-striations like rhabdomyoblasts (Wang, 2012).

Furthermore, ARMS typically harbors a chromosomal translocation, PAX3 or PAX7/FOXO1 (FKHR), rendering ARMS to be grouped as fusion positive and ERMS as fusion negative. Although ARMS rarely involves the inactivation or mutations of p53 (TP53), ERMS has a relatively high frequency of mutations or aberrations in the p53/MDM2 axis (Felix et al., 1992). In addition, metastases in ERMS show high levels of p53 mutations (Leuschner et al., 2003). The *KRAS* oncogene has been implicated in various cancers, with up to one-third of RMS cases involving the activation of one of the three RAS isoforms, including K-RAS (Ku et al., 2017; Stratton et al., 1989; Wilke et al., 1993).

Although rapid progress has been made in the field of RMS, most research has been based on xenotransplantation using human cell lines into immunodeficient models. These xenograft models do not recapitulate the complete tumor biology, including the tumor immune microenvironment. Thus, there is a significant need to develop murine RMS models that can develop and progress in immunocompetent model systems.

In this study, we developed and characterized a conditional p53 and/or K-Ras fusion-negative RMS (FN-RMS) genetically engineered mouse model (GEMM). We also performed histologic and genetic analysis of GEMM tumors, and found that these tumors had similar phenotypic and molecular features to human RMS. Based on the homologous histological and molecular traits to RMS, we believe that this mouse model, and the derived cell lines, could significantly contribute to our understanding of the molecular pathogenesis of RMS and provide valuable immunocompetent models for investigating novel therapeutic strategies for treating RMS.

## RESULTS

### Generating and characterizing non-metastatic and metastatic RMS mouse models

**Fig. 1.**
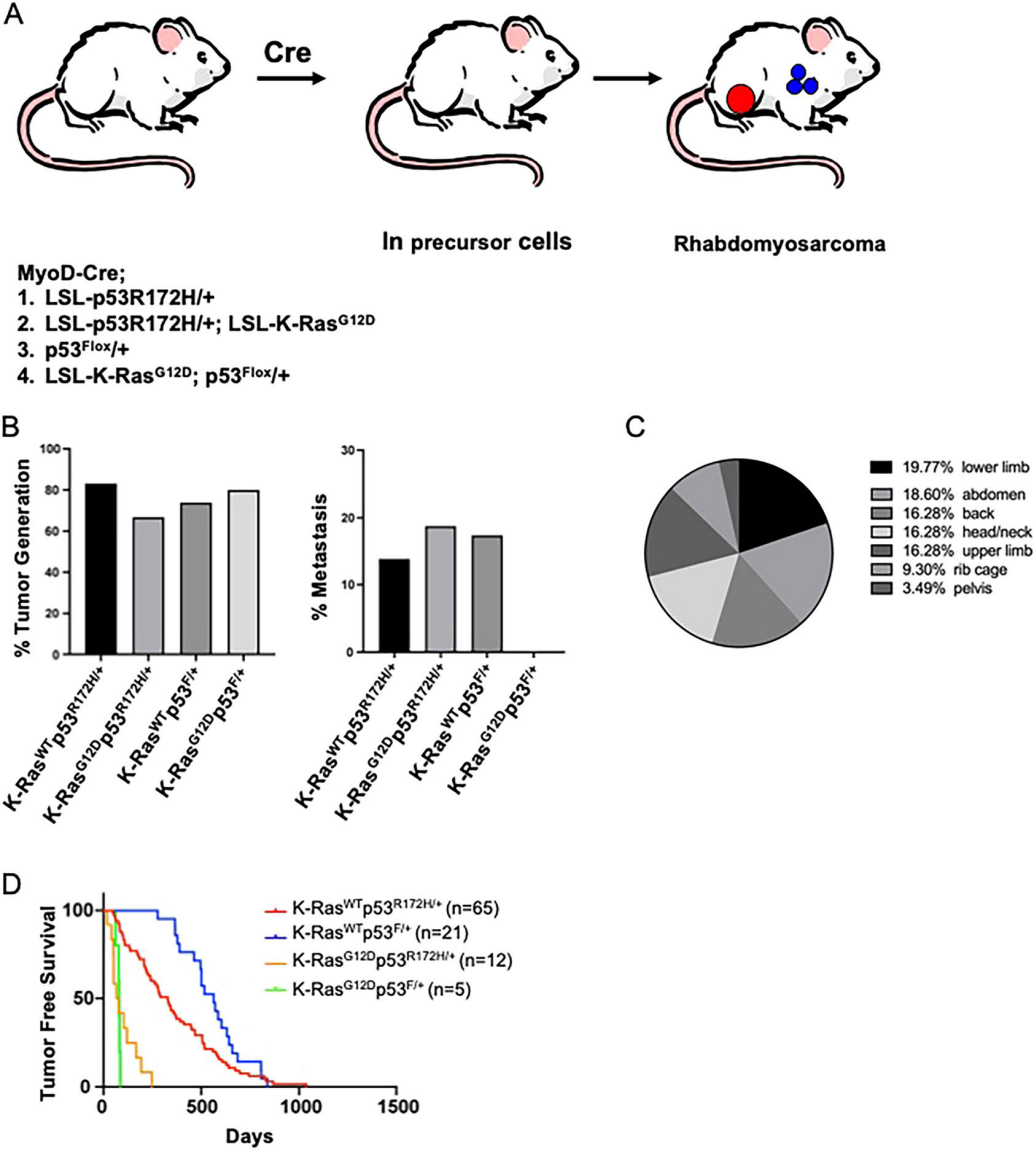
**Design and characterization of a novel genetically engineered mouse model of metastatic rhabdomyosarcoma (RMS).** (A) Schematic representing RMS-susceptible mice. All mice expressed germline MyoD-Cre allele in combination with (1) heterozygous deletion of p53 alone (F/+), (2) LSL-p53^R172H^ gain-of-function mutant p53 (R/+), (3) LSL-K-Ras^G12D^ gain-of-function K-Ras mutant in combination with heterozygous deletion of p53 (K-Ras; F/+) or (4) LSL-K-Ras^G12D^ gain-of-function K-Ras mutant in combination with LSL-p53^R172H^ gain-of-function mutant p53 (KRas; R/+). (B) Tumor incidence frequency (%) for each genotype and frequency (%) of metastatic RMS varies according to p53 genotype and K-Ras mutation. F, floxed p53; R, LSL-p53^R172H^; ‘+’, WT p53 allele. (C) Anatomical distribution of primary tumor location for RMS of the different K-Ras and p53 genotypes. (D) Kaplan–Meier plots for each of four different genotypes of mice.

To develop a RMS GEMM, we crossed MyoD (MYOD1)-Cre mice with germline floxed p53, Lox-Stop-Lox (LSL) p53^R172H^ or K-Ras^G12D^ alleles. Four distinct MyoD-Cre transgenic genotypes were monitored for tumor incidence: K-Ras-wild type (WT) mice with floxed p53 allele/WT p53 allele or LSL-p53^R172H^ allele/WT p53 allele (K-Ras^WT^p53^F/+^ and K-Ras^WT^p53^R172H/+^) and K-Ras-mutated mice with floxed p53 allele/WT p53 allele or LSL-p53^R172H^ allele/WT p53 allele (K-Ras^G12D^p53^F/+^ and K-Ras^G12D^p53^R172H/+^) (Fig. 1A).

During necropsy, we assessed for metastatic lesions in all of the major organs, noting lungs to be the most frequent site of metastasis, which we noted ranged between 10% and 20% of the animals, while there seemed to be no significant difference in incidence of tumor generation between each group (Fig. 1B). The sites of RMS development varied, with the lower limbs showing the highest frequency of lesions (19.8%), followed by abdomen (18.6%), back, head and neck, and upper limbs (16.3% each) (Fig. 1C). These results suggest that murine RMS could develop in multiple anatomical locations that are comparable to those seen in human patients.

We examined the overall survival of these genetically engineered mice, comparing the survival rates of K-Ras-mutated and K-Ras-WT mice (Fig. 1D). We noted that K-Ras-WT mice (K-Ras^WT^p53^F/+^ and K-Ras^WT^p53^R172H/+^) lived significantly longer than K-Ras-mutated mice (K-Ras^G12D^p53^F/+^ and K-Ras^G12D^p53^R172H/+^) (*P*<0.0001). In addition, there was a significant survival difference between K-Ras^WT^p53^F/+^ and K-Ras^WT^p53^R172H/+^ (*P*=0.0269) mice, but there were no statistically significant differences between the K-Ras^G12D^p53^F/+^ and K-Ras^G12D^p53^R172H/+^ cohorts (*P*=0.58).

### Immunohistochemical (IHC) analysis of GEMM tumors

**Fig. 2.**
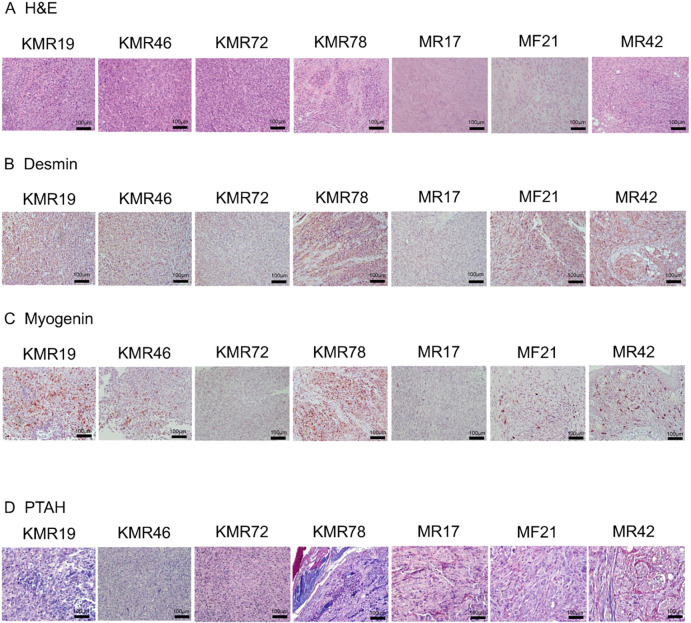
**Tumor histopathological analysis of genetically engineered mouse model (GEMM) RMS tumors.** (A) Representative H&E staining for GEMM tumors. (B) Desmin immunohistochemical (IHC) staining of GEMM tumors. They were analyzed by Hematoxylin and desmin. (C) Myogenin IHC staining of GEMM tumors. They were analyzed by Hematoxylin and myogenin. (D) Phosphotungstic acid–Hematoxylin (PTAH) staining of GEMM tumors. Muscle is stained blue black to blue purple, connective tissue is orange pink to brownish red, fibrin and neuroglia stain deep blue, and coarse elastic fibers show as purple. Scale bars: 100 μm.

Following the evaluation of tumor incidence, primary tumor development and metastatic potential in this murine model, we next sought to characterize the tumors histologically. Tumor morphology was evaluated by a veterinary pathologist with experience in mouse sarcoma models, and skeletal muscle differentiation was evaluated using phosphotungstic acid–Hematoxylin (PTAH) histochemical staining and immunohistochemistry for desmin and myogenin, markers of muscle and skeletal muscle differentiation (Fig. 2A-D). Primary tumors were moderately to poorly differentiated sarcomas, typically with round to spindloid cells with eosinophilic cytoplasm and pleomorphic nuclei (Fig. S1). Most tumors had identifiable elongated ‘strap’ cells or cells with visible cross striations that are found in RMS.

### Molecular characterization

**Fig. 3.**
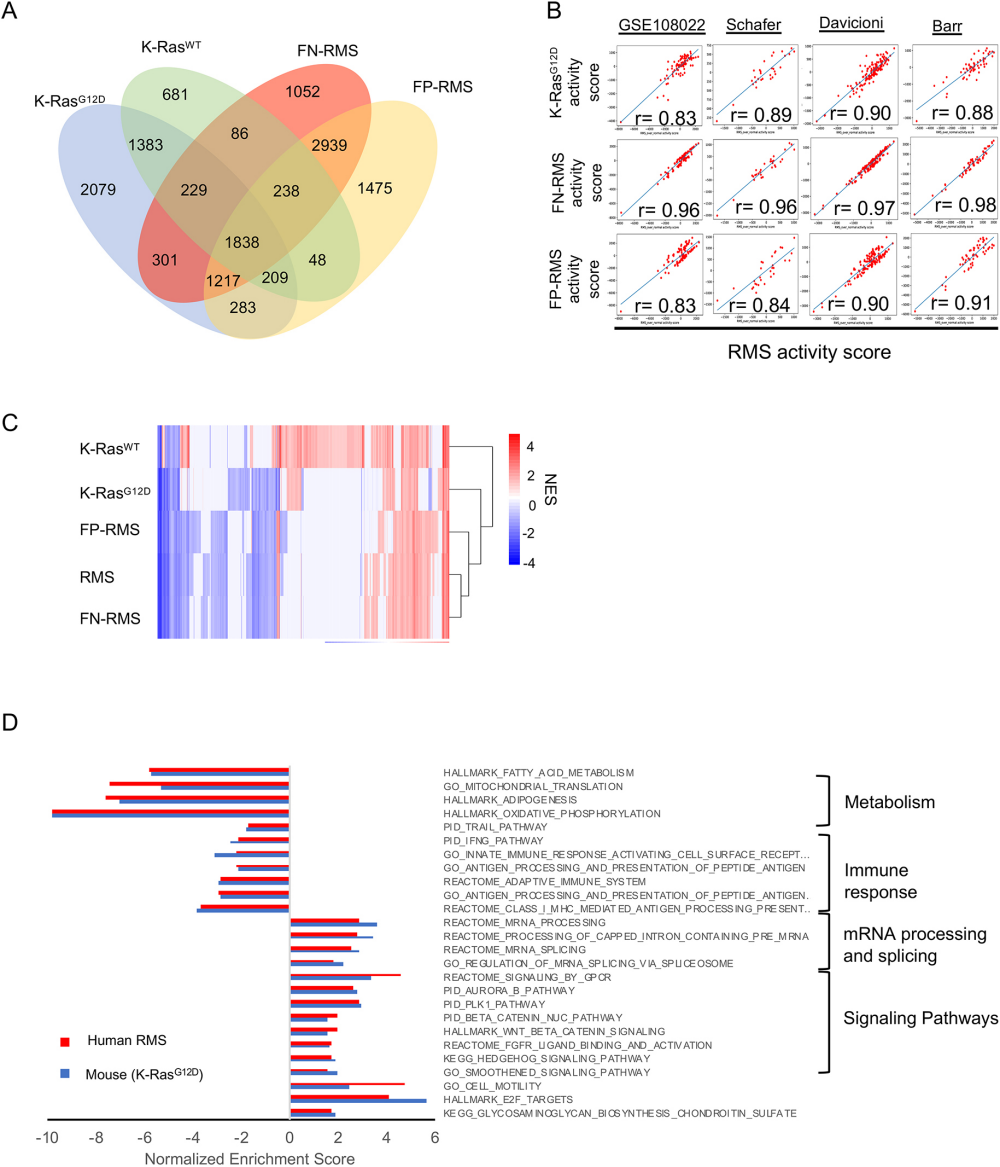
**Molecular characterization of RMS GEMM tumors.** (A) Overlap genes with human fusion-positive (FP-RMS) and fusion-negative (FN-RMS) gene signatures. (B) Pearson correlation coefficients using GSE108022, and the Schäfer (Wachtel et al., 2004), Davicioni (Davicioni et al., 2006) and Barr (GSE66533) datasets. (C) Gene set enrichment analysis (GSEA) using the mouse and human differentially expressed gene signatures. NES, normalized enrichment score. (D) Normalized enrichment scores compared between K-Ras^G12D^ and human RMS.

We performed RNA sequencing (RNA-seq) from primary GEMM tumors to examine whether gene expression in tumors from our model overlapped with gene expression changes seen in human RMS. For our studies, we used three K-Ras^G12D^ tumors (p53^R172H/+^), three K-Ras^WT^ tumors (two p53^R172H/+^and one p53^F/+^) and normal gastrocnemius muscle tissue from three non-tumor bearing mice as controls. We generated gene signatures of at least 1.5-fold change and a false discovery rate (FDR)≤0.05 for K-Ras^G12D^ or K-Ras^WT^ compared to normal murine gastrocnemius muscle tissue, as well as human RMS (either fusion negative or fusion positive) to normal muscle tissue. To determine the extent of overlap between the differentially expressed genes (DEGs) from the K-Ras^G12D/+^ and K-Ras^WT^ tumors with human RMS, we compared the murine DEGs to the human fusion-positive RMS (FP-RMS) and FN-RMS gene signatures. Of the 7900 FN-RMS DEGs, 30% (2391 genes) and 45% (3585 genes) overlapped with our K-Ras^WT^ and K-Ras^G12D^, respectively (Fig. 3A). Similarly, the 8247 FP-RMS DEGs had a 28% (2333 genes) overlap with K-Ras^WT^ and 43% (3547 genes) overlap with K-Ras^G12D^ (Fig. 3A).

We further asked whether the overlapping gene changes were consistent across multiple human RMS datasets. We performed signature correlation between our human RMS gene signature derived from the Gene Expression Omnibus dataset GSE108022 and murine *Kras* gene signatures in other human RMS cohorts. Although both the K-Ras^G12D^ and K-Ras^WT^ signatures were significantly correlated with our human RMS signature in all four cohorts analyzed, the K-Ras^G12D^ signature consistently had much higher Pearson correlation coefficients, similar to those of FN-RMS and FP-RMS, compared to K-Ras^WT^ (Fig. 3B). A previous study by Blum et al. reported that a mouse model of MyoD-expressing cells with activation of oncogenic K-Ras and p53 deletion in adult mice led to the formation of undifferentiated pleomorphic sarcoma (UPS), as opposed to RMS (Blum et al., 2013), and Doyle et al. reported that p53^R172H/+^ resulted in the rapid development of UPS in the setting of an activating K-Ras mutation (Doyle et al., 2010). We therefore also examined the correlation of our mouse model signatures to UPS DEGs and found that the K-Ras^WT^ signature had a higher correlation coefficient (0.97) with the UPS gene signature compared to K-Ras^G12D^ (0.89), which had comparable correlation coefficients to UPS with human FN-RMS and FP-RMS. Taken together, these data indicate that the K-Ras^G12D^ GEMM more closely mimics the gene expression changes seen in human RMS across multiple datasets compared to the K-Ras^WT^ model, which has gene expression changes more closely related to those seen in human UPS.

To better examine whether the K-Ras^G12D^ mouse model captures human RMS pathway changes, we ran gene set enrichment analysis (GSEA) using the mouse and human DEG signatures. Interestingly, based on unbiased hierarchical clustering using the resulting normalized enrichment scores (NESs), the K-Ras^G12D^ clustered closer to human RMS than the K-Ras^WT^ (Fig. 3C), suggesting that the K-Ras^G12D^ model is more representative of the human disease than K-Ras^WT^. More importantly, both the K-Ras^G12D^ and human RMS showed suppression of gene sets related to metabolism and immune response, whereas mRNA processing, mRNA splicing and the sonic hedgehog pathway, among other oncogenic signaling cascades, were enhanced (Fig. 3D), thus indicating a recapitulation of the human phenotype at a molecular level.

Finally, we performed Connectivity Map analysis (Subramanian et al., 2017) with the top 150 upregulated K-Ras^G12D^ genes and identified multiple drugs with high reversal scores that have known effects in RMS (Table 1). Vincristine, in particular, is an approved treatment for RMS and has been used in conjunction with irinotecan in clinical trials. FGFR and MEK (MAP2K) inhibitors have also shown promise in laboratory studies (Katoh, 2016; Degirmenci et al., 2020), further supporting our overall analysis that our GEMM model has homologous molecular signatures to the human disease.

**Table 1.**
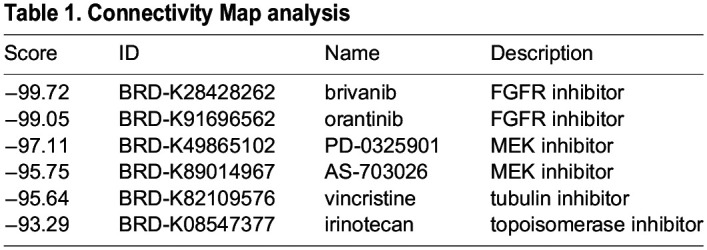
Connectivity Map analysis

### RMS phenotype is maintained in cell lines and orthotopic allografts

**Fig. 4.**
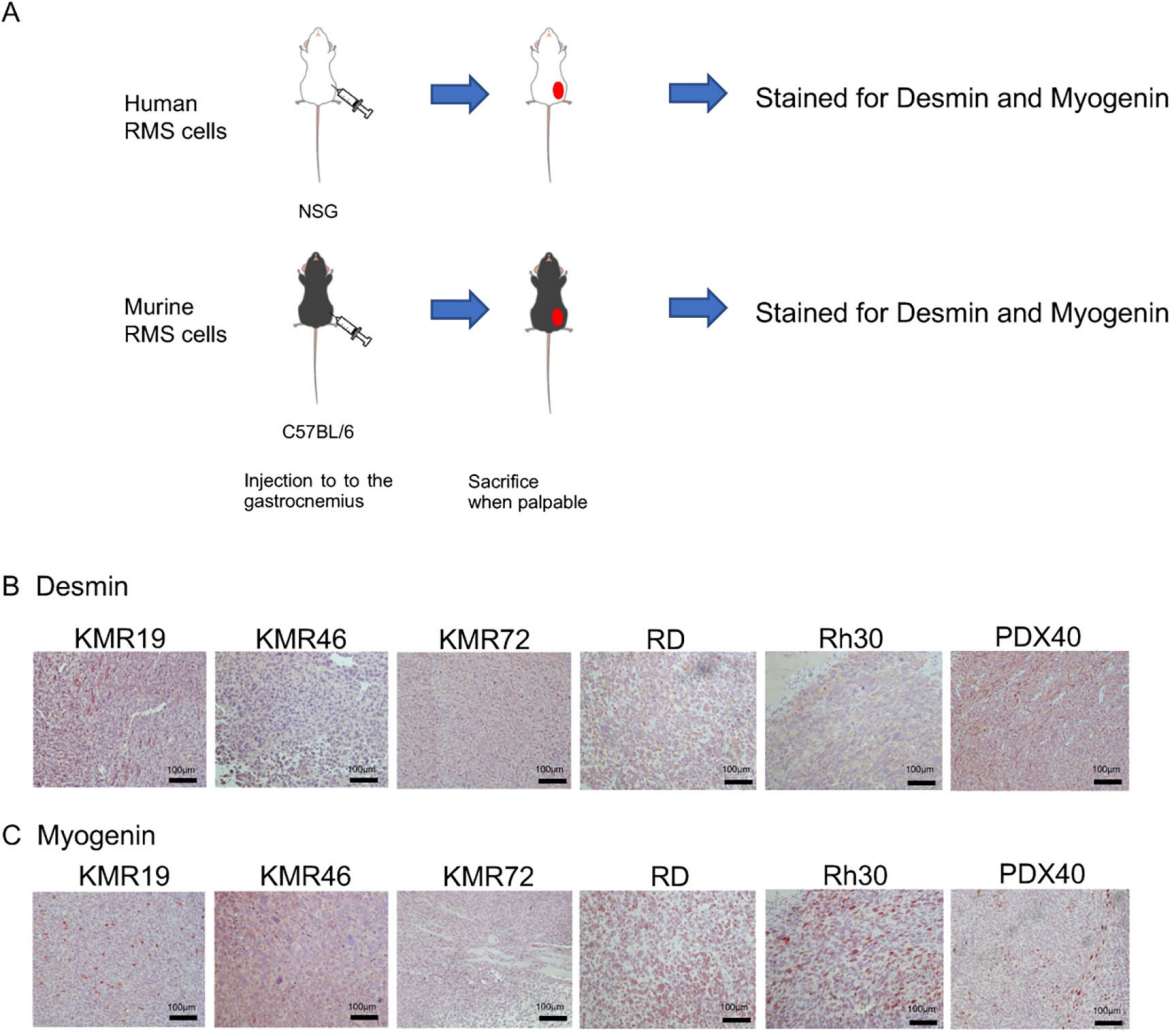
**Histopathological analysis of orthotopic transplantation into immunocompetent mice.** (A) Schema of orthotopic RMS tumor studies. The cell lines (RD, Rh30, KMR19, KMR46 and KMR72) and PDX40 (patient-derived xenograft) cells were injected into the gastrocnemius. The tumors were resected when they were palpable. (B) Desmin IHC staining of orthotopic RMS tumors. They were analyzed by Hematoxylin and desmin. (C) Myogenin IHC staining of orthotopic RMS tumors. They were analyzed by Hematoxylin and myogenin. Scale bars: 100 μm.

After isolation of GEMM tumors, as outlined in our methods, we developed primary cell lines from several GEMM tumors. We performed RNA-seq analysis on four cell lines (KMR19, KMR46, KMR72 and KMR78) and subsequently performed GSEA analysis on the DEGs between our cell lines and normal gastrocnemius muscle tissue. RNA splicing, mRNA processing and signaling pathways were consistent with the results from our K-Ras^G12D^ tumors and human tumor analysis (Fig. S2). Not unexpectedly, immune-related pathways were not consistent between our K-Ras^G12D^ tumors and cell lines. We further assessed for the ability of these cell lines to generate tumors *in vivo* by injecting 1 million cells for each K-Ras^G12D^ cell line, KMR19 (p53^R172H/+^), KMR46 (p53^F/+^) and KMR72 (p53^R172H/+^), into the gastrocnemius of C57BL/6J mice (Fig. 4A), with primary tumor growth curves shown in Fig. S3. Resulting orthotopic tumors were resected, stained for desmin and myogenin, and examined histologically, which demonstrated their resemblance to their primary GEMM tumors (Fig. 4B,C). In addition, for comparative purposes, we also stained orthotopic human tumor models for desmin and myogenin derived from RMS cell lines RD and Rh30 as well as from an institutional RMS patient-derived xenograft (PDX), TCCC-RMS40 (PDX40) (Fig. 4B,C). The staining patterns observed were again consistent with RMS, showing a high correlation between GEMM and human tumors. Of note, we attempted to develop syngeneic orthotopic tumors from K-Ras^WT^ p53^R172H/+^ (MR17 and MR42) and p53^F/+^ (MF21) in C57BL/6 mice (Table S1), but, after several months (2-4 months), no tumors were evident upon necropsy of the animals.

### Pathology of metastatic tumors from murine cell lines

**Fig. 5.**
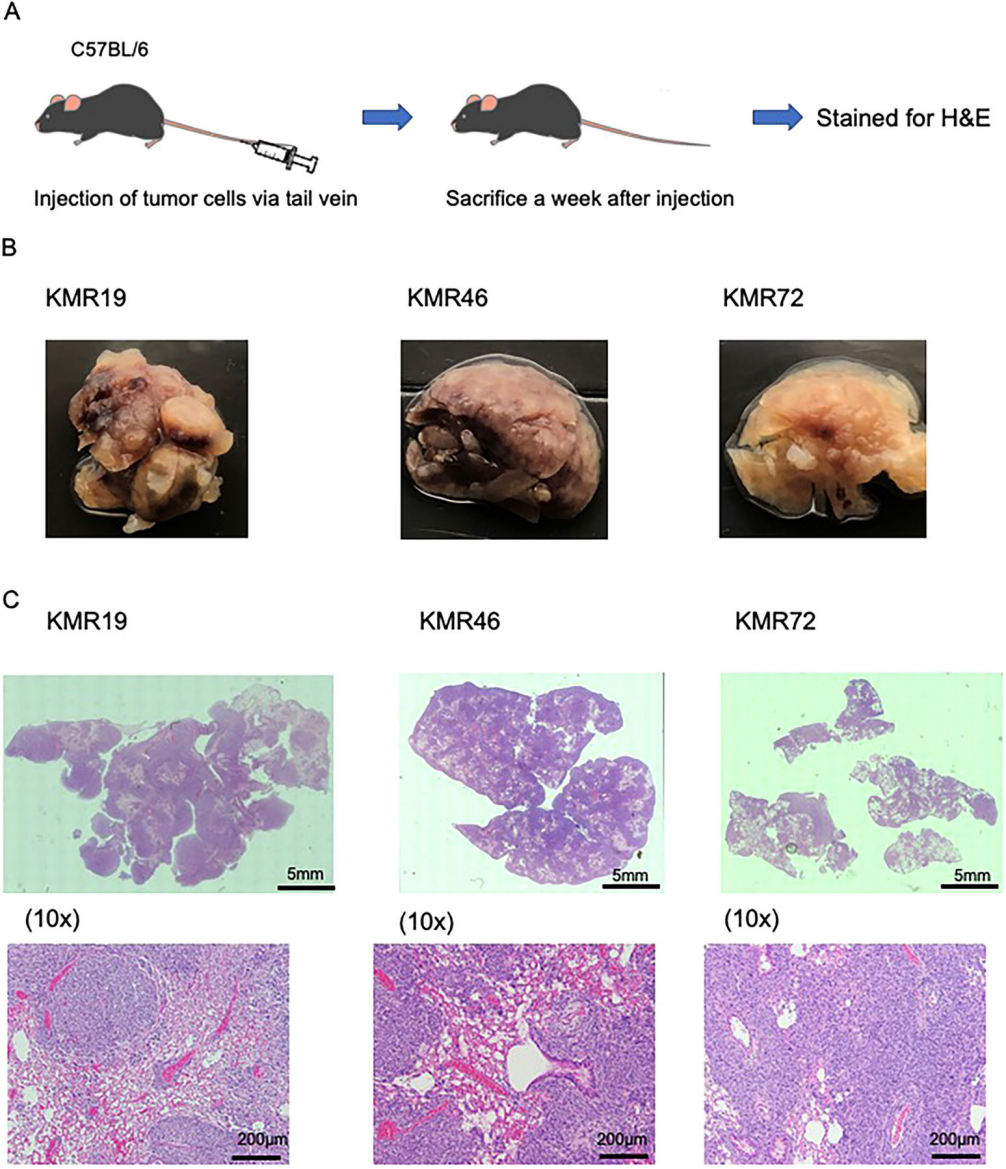
***In vivo* metastatic potential of GEMM-derived RMS cell lines in immunocompetent mice.** (A) One week post-tail vein injection, animals were sacrificed. (B,C) Brightfield images of whole lungs were obtained using tile scans (as described in the Materials and Methods) (B), and H&E staining of lung lesions was performed for lung sections (C), demonstrating a large number of metastatic lesions present. Whole lung and 10× magnification are shown. Scale bars: 5 mm (whole lung) and 200 μm (10x).

To assess the metastatic potential of these murine cell lines, KMR^G12D^ cell lines were injected via the tail vein into three C57BL/6J mice. One to two weeks following injection, mice were sacrificed and examined for metastatic disease. We observed that all mice showed evidence of macroscopic lung metastasis, which was further analyzed by Hematoxylin and Eosin (H&E) staining (Fig. 5). No metastases were observed in other organs.

### Cross-species comparison of RMS cell line models for sensitivity to anti-cancer agents

**Fig. 6.**
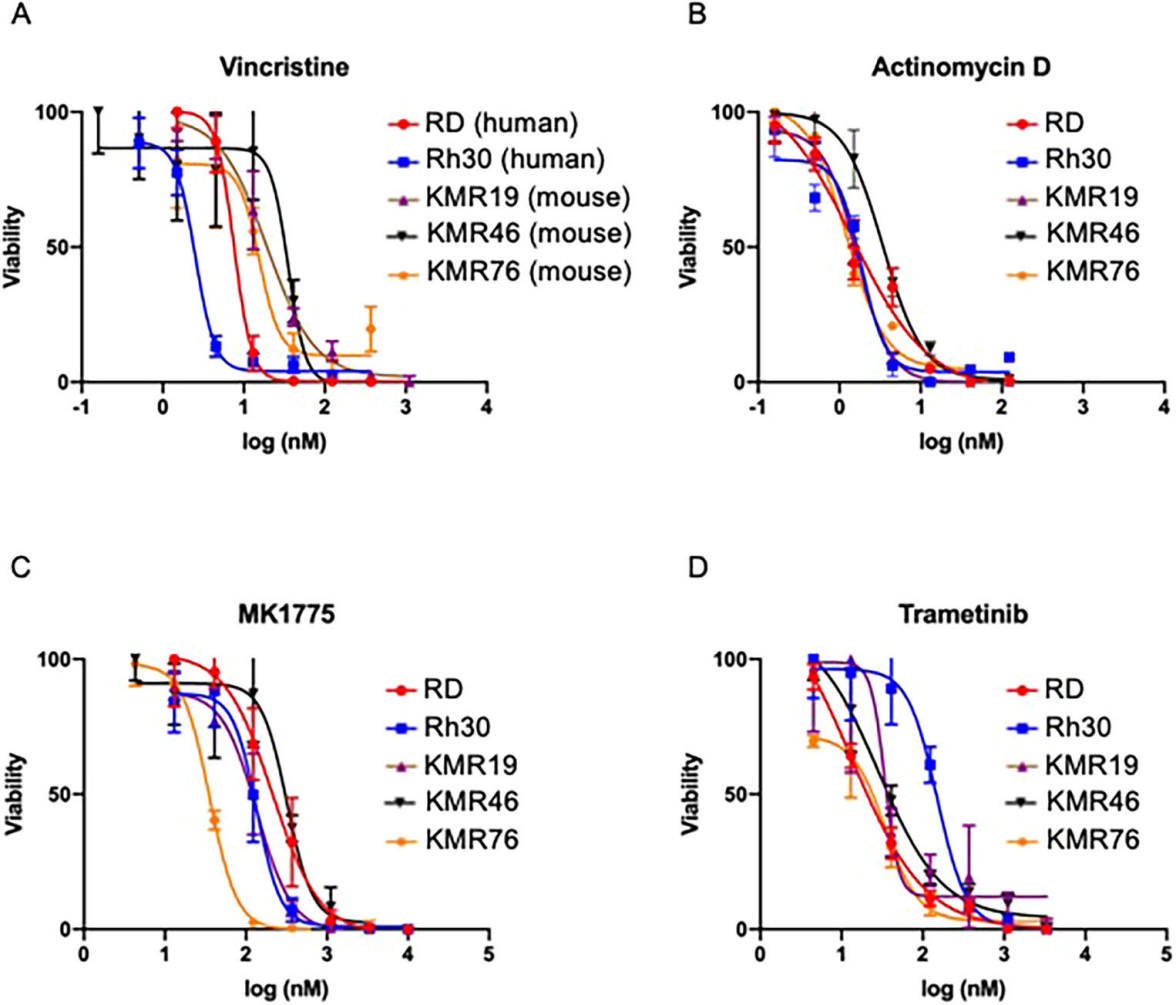
***In vitro* assessment of therapeutic responsiveness of murine RMS cell lines.** (A-D) Comparison analysis of cytotoxicity for human (RD and Rh30) and murine (KMR19, KMR46 and KMR78) cell lines for vincristine (A), actinomycin D (B), MK1775 (C) and trametinib (D). Viability was measured by CCK-8 assay, and the IC_50_ of each agent is shown in Table 2. Data presented as mean±s.d.

Based on our Connectivity Map results (Table 1), we sought to determine whether anti-cancer drugs commonly used to treat human RMS were effective treatments for GEMM-derived RMS disease. We performed cytotoxicity assays to assess cell viability in tumor-derived cell lines treated with vincristine and actinomycin D. We evaluated chemosensitivity for three murine cell lines, KMR19, KMR46 and KMR78, which were established from K-Ras^G12D^ murine tumors, as well as human RMS cell lines, RD and Rh30. Besides the aforementioned chemotherapy, we investigated targeted anti-cancer agents, including a Wee1 inhibitor, MK-1775, and the MEK inhibitor trametinib. Our rationale for investigating these latter targets include prior reports demonstrating that Wee1 is a viable therapeutic target, in combination with chemotherapy, in pre-clinical models of RMS (Stewart et al., 2017; Yohe et al., 2019), while MEK inhibition has been shown to have anti-tumor efficacy in pre-clinical FN-RMS models (Marampon et al., 2006; Yohe et al., 2018). Our findings revealed that all chemotherapeutic agents were effective in treating the murine cell lines, with highly comparable half-maximal inhibitory concentration (IC_50_) values between our murine RMS lines and the human RMS cell lines (Table 2 and Fig. 6). Overall, these *in vitro* pre-clinical studies provide evidence that our RMS GEMM-derived models are extremely valuable resources to evaluate and test novel therapeutic regimens for RMS.

**Table 2.**
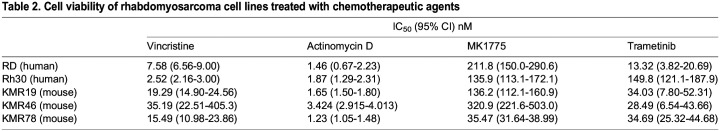
Cell viability of rhabdomyosarcoma cell lines treated with chemotherapeutic agents

## DISCUSSION

Owing to a limited availability of pediatric patient tumor samples, GEMMs and derived cell lines offer valuable resources for gaining insights into the molecular pathogenesis of tumor development and progression as well as immunocompetent pre-clinical models for evaluating the efficacy of targeted mono- and combination therapies, including immune-modulatory agents.

Through *in utero* conditional perturbations of K-Ras and p53 in skeletal muscle precursor cells, we have established a novel GEMM of RMS that exhibits high penetrance and development of histologically analogous primary tumors in homologous anatomical sites seen in RMS patients. Cross-species molecular analysis of our GEMM with human RMS transcriptomic data revealed significant overlap among key human RMS gene ontology and signaling pathways that emphasize critical molecular events driving RMS development. Furthermore, a subpopulation of our GEMM demonstrated evidence of metastatic disease, thus providing additional resources for analysis of the pathogenesis of metastatic disease. Interestingly, we did not see evidence of metastatic disease for the K-Ras^G12D^/p53^F/+^ mice; although we do not have a definitive explanation for this observation, it could be due to the rapid tumor growth requiring early euthanasia before metastatic disease is noted. In the future, we can consider resecting the primary tumor and continue to monitor and evaluate for metastatic disease. Furthermore, molecular analyses of the metastatic lesions are actively ongoing and beyond the scope of this present study. We did note some limitations to these studies, including observing occasional multifocal disease in some mice, which is rare in RMS.

Although there have been previous FN-RMS murine models reported (Hettmer et al., 2011; Rubin et al., 2011; McKinnon et al., 2015), our model differs from those through *in utero* genetic perturbations that drive endogenous and spontaneous development of RMS. In these models, we were able to observe spontaneous metastatic tumors in the lung without venous injection and isolate syngeneic K-Ras mutated cell lines, which helps us to investigate murine RMS. Interestingly, prior reports of various skeletal precursor conditional RMS models have demonstrated discrepancy between PAX7- and MyoD-driven recombinase. Blum et al. used PAX7-CreER and MyoD-CreER mice to transform Pax7^+^ and MyoD^+^ myogenic progenitors by expressing oncogenic K-Ras^G12D^ and deleting p53 *in vivo*, and found that PAX7-CreER mice developed RMS and UPS, whereas MyoD-CreER mice developed UPS (Blum et al., 2013). However, one caveat for the use of prior MyoD-Cre models with p53 and K-Ras alterations, which generated more undifferentiated soft tissue sarcomas, was the induction of the recombination events after birth of the animals. It has been reported that there are potential differences in myogenic promoter activity that appear to be associated with *in utero* versus neonatal and older mice (Rubin et al., 2011). Thus, our findings that *in utero* induction of p53 and K-Ras perturbations lead to histological and molecular signatures consistent with RMS could be attributed to this facet of developmental biology. However, additional studies are essential to further probe these features, which will help delineate the molecular facets contributing to this disease spectrum.

Interestingly, analysis of the p53-only altered tumors demonstrated signatures were more consistent with human UPS (Tsumura et al., 2006; Doyle et al., 2010), while the knock-in of the mutant K-Ras not only significantly accelerates tumor formation, but also drives the molecular signatures that highly correspond to human FP-RMS and FN-RMS. Therefore, our models can potentially be used to gain additional insights into the critical molecular signatures differentiating this spectrum of soft tissue sarcomas.

Besides providing valuable models to gain insights into the molecular events that drive RMS initiation and progression, our GEMM is extremely valuable secondary to the tumor-derived cell lines. As we have preliminarily demonstrated, a significant advantage of these murine RMS cell lines is that they allow for highly efficient syngeneic orthotopic tumor development in immunocompetent mice. Furthermore, upon venous injection, these cell lines are also capable of rapidly establishing metastatic lung disease, thus providing additional resources for the evaluation of molecular and pharmacological targeting of primary, and metastatic, disease. Finally, we provide additional evidence that the murine RMS cell lines can be surrogate, and highly complementary, models for evaluation of targeted and chemotherapy secondary to their very comparable responsiveness to human cell lines for various therapies, including mimicking enhanced sensitivity of the *RAS* mutant RD human cell line to the MEK inhibitor trametinib.

Ongoing and future directions include the utilization of these models to further dissect molecular mechanisms of RMS metastasis, characterization of the primary and metastatic tumor immune microenvironment, *in vivo* studies further probing identified metastatic signatures, and small molecules not only targeting critical tumor intrinsic signaling pathways but also immunomodulatory agents using these immunocompetent models.

In conclusion, we have created a novel GEMM of RMS with metastatic capabilities and derived tumor model resources that can be utilized for further molecular and therapeutic studies necessary to developing more effective treatment regimens for RMS patients.

## MATERIALS AND METHODS

### Cell lines

Human RMS cell lines (RD and Rh30) were purchased from American Type Culture Collection (Manassas, VA, USA). The cells were grown and maintained in RPMI-1640 medium (Thermo Fisher Scientific, Waltham, MA, USA) supplemented with 10% fetal bovine serum (FBS; Thermo Fisher Scientific) and 1% penicillin/streptomycin (Thermo Fisher Scientific), and cultured in a humidified 5% CO_2_ incubator at 37°C.

### Mice

Transgenic mouse lines were generated through MyoD promoter-driven Cre recombinase with core enhancer, which directs the early activation of MyoD, as reported by Chen et al. (2005). These mice were backcrossed using the C57BL/6J strain and further crossed to either floxed p53 mice, LSL-p53^R172H^ mice or K-Ras^G12D^ mice. Both male and female mice were used. Genotyping was performed using PCR. All research was performed in compliance with the Baylor College of Medicine IACUC (Animal Protocol AN-5225) and AAALAC recommendations as published in The Guide for the Care and Use of Laboratory Animals (NRC1996). All animals were monitored closely and underwent comprehensive necropsies following humane euthanasia at an endpoint. All major organs and tumors were evaluated, and tissues suspected of metastatic disease were submitted for histological analysis.

### Murine cell lines

Murine RMS cell lines (KMR19, KMR46, KMR72 and KMR78) were isolated from primary K-Ras-mutated RMS tumors. Resected tumors were diced and dissociated using a Tumor Dissociation Kit, mouse (Miltenyi Biotec, Cologne, Germany) and a gentleMACS Dissociator (Miltenyi Biotec). The dissociated cells were resuspended and filtered through a 70 μm cell strainer (Corning), then seeded, established and subcultured. The established cell lines were cultured in RPMI-1640 medium (Life Technologies, Carlsbad, CA, USA) supplemented with 1% penicillin/streptomycin and 10% FBS, and cultured in a humidified 5% CO_2_ incubator at 37°C. All murine RMS cell lines are available upon request from the corresponding author (J.T.Y.).

### Tumor injection

KMR19, KMR46, KMR72, KMR78, RD, Rh30 and PDX40 cells were harvested and resuspended at a concentration of 1×10^6^ cells per 25 μl PBS and then mixed with 25 μl Matrigel matrix (Corning, Corning, NY, USA). The cell–Matrigel mixture was subsequently injected into the gastrocnemius of C57BL/6J mice (*n*=3/cell line), and the tumors were resected once palpable.

KMR19, KMR46, KMR72 and KMR78 cell lines were harvested and resuspended at a concentration of 1×10^6^ cells per 200 μl PBS. The cells were injected into the tail vein of the mice (*n*=3/cell line), which were subsequently sacrificed a week after injection. Cell lines were authenticated using short tandem repeat analysis and frequently checked for mycoplasma contamination.

### Cell viability assay

To assess cell viability, cells were seeded at 5×10^3^ cells per 96-well plate (Corning) for 24 h and then incubated with various concentrations of vincristine, irinotecan, SN38, MK1775 and trametinib (Selleck Chemicals, Houston, TX, USA) for 72 h. Subsequently, the degree of cell viability was evaluated using the Cell Counting Kit-8 (CCK-8; Dojindo, Kumamoto, Japan) according to the manufacturer's protocol.

### Immunohistochemistry

GEMM and orthotopic model-derived xenograft tumors, as well as clinical specimens, were embedded in paraffin, fixed, and analyzed by Hematoxylin, Eosin, myogenin (1:300; EPR4789, polyclonal; Abcam, Cambridge, UK) and desmin (1:300; PA5-16705, polyclonal; Waltham, MA, USA) staining. For IHC analysis, a VECTASTAIN ABC Kit, Rabbit IgG (Vector Laboratories, Burlingame, CA, USA) was used, and the staining was visualized using a Vector NovaRED Peroxidase Substrate Kit (Vector Laboratories).

### PTAH staining

For PTAH staining, we used a Rapid PTAH Stain Kit (Polysciences, Warrington, PA, USA). Briefly, tumor sections slides were placed in Langeron's iodine after deparaffinization and rehydration, and then transferred to 5% sodium thiosulfate. After rinsing in water, slides were stained in pre-heated PTAH.

### Imaging

Images of H&E and IHC staining were obtained using a BX53 Biological Microscope (Olympus, Tokyo, Japan). The whole pictures of lung sections were obtained by tile scanning, which merged 20× images and allowed the acquisition of overview images of the specimen, and Leica DMi8 and LAS X software (Leica Microsystems, Wetzlar, Germany).

### RNA-seq library preparation and sequencing

RNA samples underwent quality control assessment using the RNA tape on Tapestation 4200 (Agilent Technologies) and were quantified using a Qubit Fluorometer (Thermo Fisher Scientific). The RNA libraries were prepared and sequenced at the University of Houston Seq-N-Edit Core per standard protocols. RNA libraries were prepared with QIAseq Stranded Total RNA Library Kit (Qiagen) using 500 ng input RNA. mRNA was enriched with Oligo-dT probes attached to Pure mRNA beads (Qiagen). RNA was fragmented, reverse transcribed into cDNA, and ligated with Illumina sequencing adaptors. The size selection for libraries was analyzed using the DNA 1000 tape Tapestation 4200 (Agilent Technologies). The prepared libraries were pooled and sequenced using NextSeq 500 (Illumina), generating ∼17million 2×76 bp paired-end reads per samples.

### Total RNA-seq analysis

Paired-end sequencing reads were trimmed using trimGalore software. The trimmed reads were mapped using STAR against the murine genome build UCSC mm10 and quantified using featureCounts (Liao et al., 2014) against the Gencode gene model. Human RMS gene signatures were derived using the RNA-seq counts data for GSE108022, which includes 101 RMS (35 FP-RMS and 66 FN-RMS) samples and five normal muscle samples. Differential expression analysis was performed using DESeq2 R package (1.28.1) (Love et al., 2014). *P*-values were adjusted with Benjamini and Hochberg's approach for controlling the FDR. Significantly differentiated genes between the comparisons were identified by applying the criteria of adjusted *P*-value<0.05 and fold change exceeding 1.5×. Pathway enrichment analysis was carried out using the GSEA (http://software.broadinstitute.org/gsea/index.jsp) software package; significance was achieved for adjusted *q*-value<0.25.

Signature correlations were performed using normalized expression data in the Schäfer (*n*=30; Wachtel et al., 2004), Davicioni (*n*=147; Davicioni et al., 2006) and Barr (*n*=58; GSE66533) datasets in R2:Genomics Analysis and Visualization Platform (http://r2.amc.nl). The UPS cohort RNA-seq counts data were kindly provided by Dr Nischalan Pillay (Steele et al., 2019) and consisted of 58 tumor samples and five normal adjacent tissue controls. For each cohort, a *z*-score was computed for each gene in a given gene signature per patient sample resulting in a patient activity score. For each combination of signatures, the individual patient activity scores were plotted on the *x*- and *y*-axes, and the Pearson correlation coefficient along with *P*-value were calculated.

### Statistical analysis

*P*<0.05 was considered statistically significant. All statistical analyses were performed using Prism (GraphPad Software, San Diego, CA, USA).

## Supplementary Material

Supplementary information
