# Supplementary material for: K-Ras and p53 mouse model with molecular characteristics of human rhabdomyosarcoma and translational applications
Source: Dis Model Mech. 2022 Feb 17;15(2):dmm049004. doi: 10.1242/dmm.049004 (PMC8844455; doi:10.1242/dmm.049004)
Supplement: Supplementary information [file dmm-15-049004-s1.pdf]

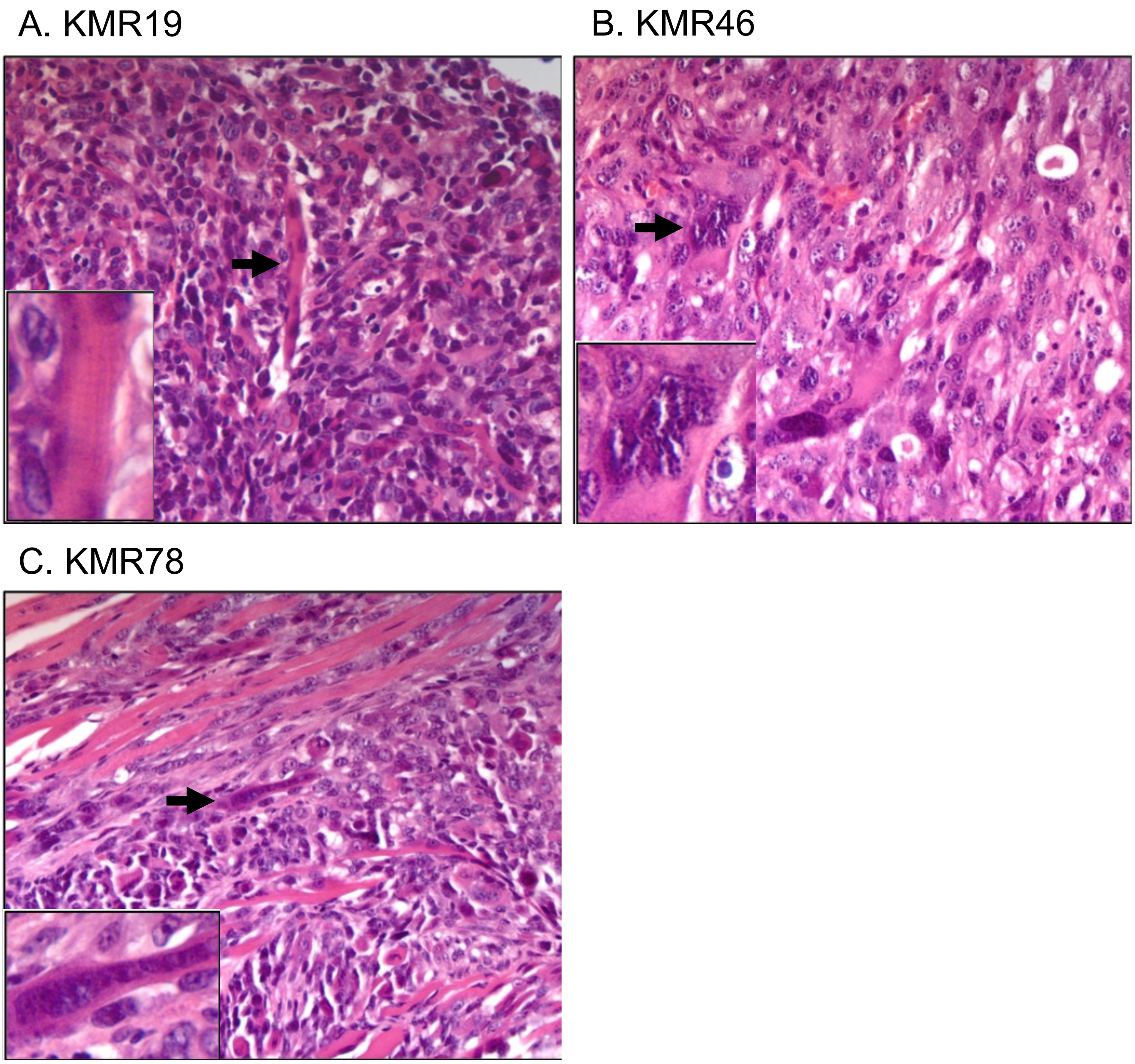

Fig. S1. Histologic features of KMR rhabdomyosarcomas

(A) Rhabdomyosarcoma with elongated multinucleate strap cell and cross striations (arrow and inset). (B) Anaplastic, poorly differentiated rhabdomyosarcoma with large, irregular nuceli (arrow and inset). (C) Rhabdomyosarcoma with elongated, multinucleate strap cell (arrow inset) invading preexisting benign skeletal muscle.

Disease Models & Mechanisms • Supplementary information

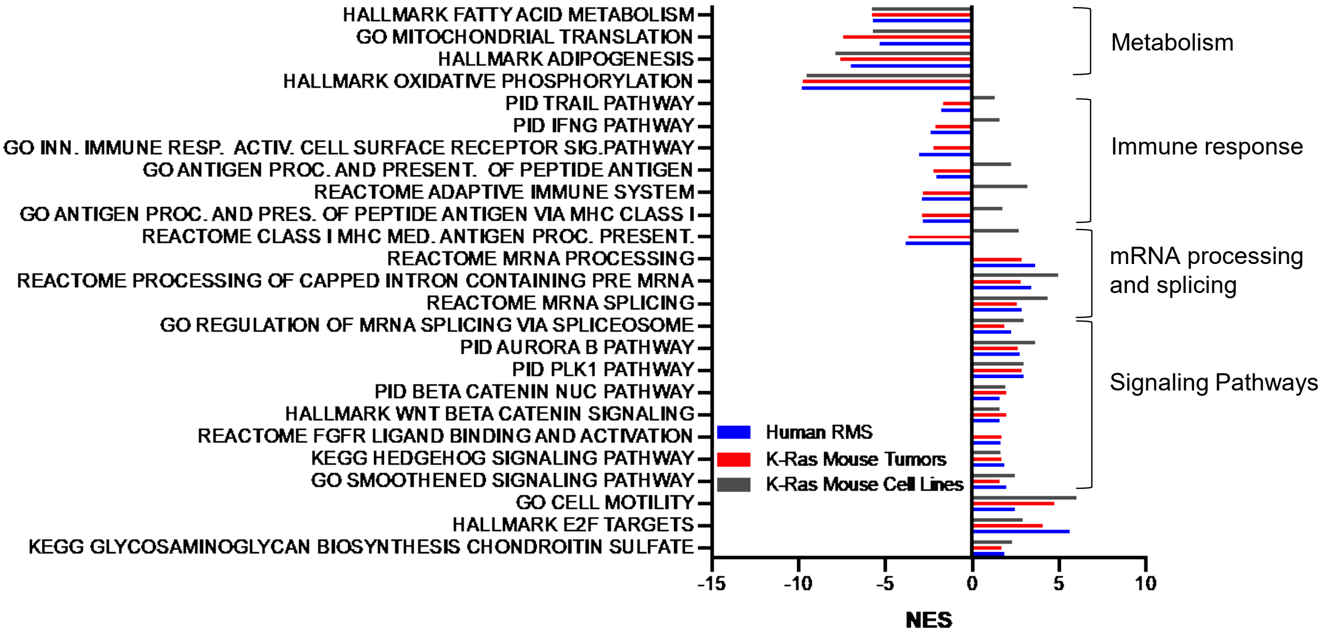

Fig. S2. GSEA comparisons of human patient tumors to murine tumors and cell lines

Normalized Enrichment Scores for Gene Set Enrichment Analysis (GSEA) performed using the K-Ras^{G12D} murine tumor (red), K-Ras^{G12D} murine cell lines (black) and human RMS tumor (blue) DEGs. 'NES'= normalized enrichment score.

Disease Models & Mechanisms • Supplementary information

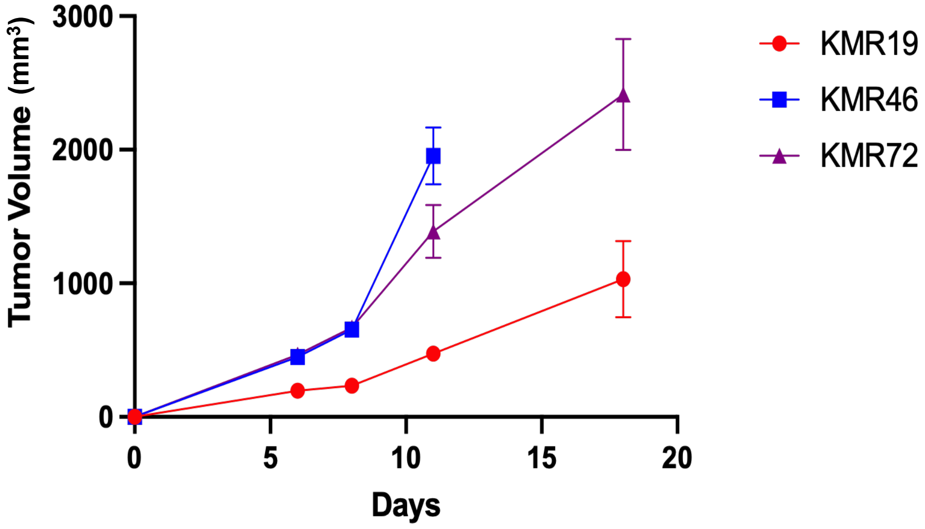

Fig. S3. Growth curve of KMR^{G12D} cell lines.

1x 10^6 cells from the KMR19, KMR46 and KMR72 cell lines were injected into the gastrocnemius of C57BL/6J mice (n=3), and tumors were monitored and measured. Tumor volume was calculated by the following formula: V (mm^3) = $0.5 \times X \times Y^2$, where X is the longest tumor axis and Y is the shortest tumor axis. Data presented as mean ± SD.

Table S1. List of the models and genotypes

Models	Genotype
KMR19	$Kras^{G12D}$ $p53^{R172H/+}$
KMR46	$Kras^{G12D}$ $p53^{F/+}$
KMR72	$Kras^{G12D}$ $p53^{R172H/+}$
KMR78	$Kras^{G12D}$ $p53^{R172H/+}$
MR17	$Kras^{WT}$ $p53^{R172H/+}$
MF21	$Kras^{WT}$ $p53^{F/+}$
MR42	$Kras^{WT}$ $p53^{R172H/+}$

Disease Models & Mechanisms • Supplementary information
